# Capital sharing and socialization in an interprofessional student-led clinic: a Bourdieuan analysis

**DOI:** 10.1186/s12909-024-05117-7

**Published:** 2024-02-19

**Authors:** Chris Roberts, Priya Khanna, Annette Burgess

**Affiliations:** 1 Division of Clinical Medicine, School of Medicine and Population Health, The University of Sheffield, S10 2RX Sheffield, United Kingdom; 2 School of Clinical Medicine, Faculty of Medicine & Health, The University of New South Wales, Sydney, NSW 2052 Australia; 3 Sydney Medical School, Education Office, Faculty of Medicine and Health, The University of Sydney, Camperdown, NSW 2006 Australia

**Keywords:** Student-led clinic, Student-delivered clinic, Patient outcomes, Interprofessional education, Medical education, Social capital, Cultural capital, Interprofessional practice, Bourdieu

## Abstract

**Background:**

Interprofessional student-led clinics offer authentic clinical experiences of collaborative patient care. However, theoretical research on the sustainability of these clinics, considering forms of capital beyond the economic, remains limited. This study addresses this gap by employing Bourdieu's theoretical framework to explore how alternative conceptions of capital; both social and cultural might sustain conditions for interprofessional working in a student-led clinic serving patients living with a chronic neurological impairment.

**Methods:**

The teaching and learning focussed clinic was established in 2018 to mirror a clinical service. Semi-structured focus groups with participants involving 20 students from 5 professions and 11 patients gathered in-depth insights into their experiences within the clinic. A thematic analysis was guided by Bourdieu’s concepts of field, habitus, and capital.

**Results:**

In the complex landscape of the student-led clinic, at the intersection of a patient support group, a hospital-based aged care facility, and university-based healthcare professions, three pivotal mechanisms emerged underpinning its sustainability: Fostering students’ disposition to interprofessional care, Capitalizing on collaboration and patient empowerment, and a Culture of mutual exchange of capital. These themes illustrate how students and patients specific dispositions towards interprofessional healthcare enriched their habitus by focusing on shared patient well-being goals. Diverse forms of capital exchanged by students and patients fostered trust, respect, and mutual empowerment, enhancing the clinic experience.

**Conclusion:**

This study bridges an important gap in theoretically informed explorations of the conditions for sustaining student-led clinics, drawing on Bourdieu’s theory. It accentuates the significance of investment of diverse forms of capital in such clinics beyond the economic, whilst emphasizing a primary commitment to advancing interprofessional healthcare expertise. Recognizing patients as equal partners shapes clinic dynamics. In order for student clinics to thrive in a sustainable fashion, educators must shift their focus beyond solely maximizing financial resources. Instead, they should champion investments in a wider range of capital forms. This requires active participation from all stakeholders; faculties, patient partners, service providers, and students. These findings underscore the importance of investing in interprofessional learning by optimizing various forms of capital, and embracing patients as dynamic contributors to the clinic's sustainability.

## Introduction

Interprofessional student-led health clinics have emerged as a valuable educational strategy. They offer unique opportunities for students, patients and educators to engage in authentic, collaborative patient care. These clinics build upon the diverse knowledge, skills, and behaviours of participants, providing a practical experience of interprofessional education (IPE) [1–4]. Despite the growing global recognition of the importance of IPE [5, 6] and an expanding literature attesting to its benefits, [7] the implementation and sustainability of educationally impactful but resource-intensive activities poses significant challenges [8, 9]. Researchers have begun to recognise the need for theory informed approaches to the organisation of student-led clinics [10]. This issue is of critical concern because the implementation and sustainability of IPE heavily depend on educational and practice settings that are predominantly uniprofessional, with medicine often dominating the prevailing culture of training [11].

There remains a notable gap in theory-based empirical research to identify underlying mechanisms that sustain conditions for interprofessional and collaborative practice to flourish [12]. Existing research has identified various opportunities and barriers for sustaining student-led clinics in different contexts. There are several factors including economic, political, social-cultural, physical, and technological. Funding for student-led clinics is complex and influenced by various enablers including healthcare funding models, faculty volunteerism, strong partnerships with community organizations and stakeholders, and well-developed business plans outlining goals and financial projections [13–15]. Conversely, barriers include lack of funding availability and limited experience in fundraising and financial management, preferences to work within one’s own professions, logistical issues such as timetabling and space allocation, availability of trained facilitators or supervisors, lack of operational structure for delivering IPE activities, legal and insurance concerns around student clinical decision making, and patient recruitment and management [16–18].

In response to these challenges, student-led clinics can vary from simulating authentic clinical practice, to service provision for underserved communities with funding often associated with the latter [18–20]. Given the intricacies of establishing a student clinic, there is a need to reframe conventional economic and resource arguments, and seek alternative means to appreciate the educational benefits for students, faculty and improved health outcomes for patients. Using Bourdieu's social theory, this study explores various types of capital - social (relationships, networks), cultural (shared values, norms), and symbolic (prestige) - beyond traditional economic considerations. The opportunity for exploring this issue arose when a student-led clinic was established through collaboration between educators, healthcare faculties, an aged-care facility, and a patient support group. By applying Bourdieu's social theory, this research aimed to explore how the use of various forms of capital might serve as a mechanism influencing the sustainability of interprofessional collaboration in student clinics. Findings from the study would contribute to theoretical understanding of sustaining conditions for interprofessional and collaborative practice in student-led clinics.

### Theoretical framework: Introducing Bourdieu’s field, habitus, and the forms of capital

Bourdieu's concepts provide a multi-dimensional understanding of the clinic as a social space. At the core of Bourdieu's framework are three pivotal concepts: field, habitus, and capital, and their interrelation with power. These concepts provide a lens through which the complexities of social interactions within student-led clinics can be analyzed and understood. Bourdieu’s theory of practice examines the opportunities and constraints in overcoming cultural domination and developing reflexivity, thus holding promise for understanding the conditions for sustainability in IPE [21].

Field refers to a social domain (e.g., family, school, sport, healthcare) with distinct roles, relationships, and practices that individuals become socialized into. Each specific field is distinguished by its objective relations, its agents and institutions, and the specific logic of practice or “rules of the game.” [22] Fields coexist and overlap at various levels, with smaller fields nested within larger ones. Within a field, individuals vie for position, seek control over capital invested in the field, and may attempt to alter the rules.

Habitus encompasses the durable and transposable dispositions of agents (individuals, groups of actors or institutions) within a social setting [23]. It comprises competencies, expectations, biases, and insecurities that develop and shape their aspirations and practices. Habitus sets the boundaries of agency and influences future choices and actions. Habitus is enriched with the capital which individuals acquire as they are socialised in various domains such a family life, leisure, formal education, and work [23, 24].

Capital and Power: In addition to economic capital, Bourdieu (1997) describes three other forms of capital: social, cultural, and symbolic. Social capital encompasses shared norms, values, trust, networks, and social relations that facilitate collaboration and collective action [25]. It is influenced by an individuals’ social network, and the power within those networks [24]. Cultural capital is what one knows and what one has, for example possessing the ‘right’ kind of knowledge [26] Symbolic capital is acquired automatically upon entering a field and refers to the amount of prestige or honour derived from other forms of capital. Symbolic capital involves a good faith economy where capital exchange is based on mutual trust [22]. Symbolic power makes people see and believe in a certain vision of the world and directs them to act accordingly. Relationships between individuals depend upon the accumulation of symbolic power allowing an individual to impose their world view on others [27].

### Bourdieu’s theoretical frameworks in IPE settings

Bourdieu’s theoretical lens has been applied in both uniprofessional e.g. medicine [28–30], and interprofessional settings, [31–33] in order to understand the conditions for changing the dominating cultures and complex relationships between field, habitus, and capital. Medicine, as the dominant culture, has traditionally emphasized technical clinical competence (cultural capital) over caring, which can negatively impact the habitus of medical students [28]. Conversely, in allied health, the relationships and interactions students experienced during IPE activities, was a major influence on enriching students’ habitus [31]. Bourdieu’s theory of social space (a mapping of individuals positions in the field) has been used in the context of how the ‘nurses’ station” on hospital wards promotes communication and teamwork for the provision of safe and quality patient care [34].

### Study aim and research questions

Using Bourdieu's social theory, this paper explores how students and patients leverage diverse forms of capital (cultural, social, symbolic) within an IPE student clinic. By analyzing the relationships between field, habitus, and capital and power, we seek to uncover the underlying mechanisms that contribute to the sustainability of an interprofessional student-led clinic. Our specific research questions in this qualitative study were as follows:

1. How do students actively cultivate dispositions that contribute to interprofessional care within the student-led clinic?
2. How did patients’ expression of their habitus and contributions of capital influence collaboration within the student-led clinic?
3. What are the diverse forms of capital brought by both students and patients, and how is this capital exchanged to impact the overall culture of the student-led clinic?

## Methods

### Setting and Participants

The study took place within a teaching focussed student-led interprofessional clinic for people living with Parkinson’s disease. This has been described in detail elsewhere, [35] where 32 senior students and thirteen patients took part throughout 2018. In summary, the student clinic involved five health care professions (medicine, pharmacy, occupational therapy, physiotherapy, and speech pathology) collaborating with a patient support group and an aged care facility in a local teaching hospital to provide a simulated clinic. Students who were undertaking clinical placements as part their program volunteered for attendance at the clinic having made aware of it by their profession specific educators The clinic had been designed to emulate an existing multidisciplinary service clinic for people living with Parkinson’s disease, run within the aged care facility. However, the University took the view that students could not give their clinical judgements directly to patients. Any clinical concerns in the student-led clinic were referred to the aged care facility director. Patients noted three well-being concerns that they wanted addressing in the clinic. They rotated through four ‘stations’, spending 30 minutes at each with a pair of students from the same healthcare profession, before moving on to the next ‘station.’ Students took a history and where appropriate performed an examination. Their goal was to produce a collaborative management plan based around the three personal well-being goals provided by the patient. Further detail of the clinic are given in Table 1.

**Table 1:** Interprofessional student-led clinic for people living with Parkinson’s disease

Overview
There was no specific funding for the clinic. The program was delivered from 8.30am – 1:00pm, five times throughout the year. At each clinic, three or four real patients, six to eight students, and at least one clinical educator and one educator were in attendance. Booking for the clinic were organised by the university clinical school staff.
Clinic participants:
*Students:* Eight final year students from medicine, pharmacy and allied health (physiotherapy, occupational therapy, speech pathology) in any one clinic *Patients:* The patient volunteers from community groups for people living with Parkinson’s disease and their carers and families. They were asked to identify three areas which they hoped the clinic would focus on, for example increasing exercise, or fall prevention.
*Educators:* At each session, there was at least two clinical educators, who ran an initial orientation session (and provided supervision (pharmacy and medicine) with an aged care consultant in whose unit the clinic ran, on standby.
Clinic schedule and record keeping
Students were provided with a 30 minute orientation led by a clinical educator, and given the Parkinson’s Disease Clinic General Assessment Form to guide the patient assessment. Then in pairs matched with their own health care profession, students rotated through four ‘stations’, spending 30 minutes with each patient. Each time students met with a patient, the patient notes were passed to the next pair of students. Students then organised a interprofessional team meeting lasting 30 minutes, and then presented to the academic educator(s) (30 minutes) who gave feedback. Together, students were required to produce an integrated patient management plan to present to the supervisor academic. In this clinic the patient did not hear the management plans as the patient partners had other activities to attend.

### Data collection

To explore our research questions, qualitative data were collected from three of the five clinics that ran in 2018. A total of 20 students (6 male, 13 female) who attended one of the clinics participated in the student focus groups. Focus groups allowed for in-depth exploration of participants' perceptions and experiences through group discussion. This enabled researchers to capture not only individual viewpoints but also the interplay of ideas and perspectives within the social context of the clinic. [36] The student participants represented various professions, including medical (9), physiotherapy (5), pharmacy (2), speech (3), and occupational therapy (1). The student focus group discussions were led by AB an experienced researcher with questions exploring the student experience of the clinics including shared decision making, working in a team, health profession differences, leadership, and patient safety.

In addition, there were 11 patients (5 female, 6 male) living with Parkinson's disease who took part in the patient focus groups led by AB. All patients were taking anti-Parkinson's medication and had regular consultations with a neurologist and access to a general practitioner ( family physician). Among them, two had mild early symptoms of Parkinson's within the last 18 months, eight had moderate symptoms, and one patient had severe symptoms and relied on a wheelchair for mobility. Additionally, all of the patients were fluent in English. Patients were interviewed after a light lunch and a beverage. Interviews covered benefits and insights received on their health goals, communication with the student team, and suggestions for improving the clinic experience.

### Data analysis

Focus group data were transcribed verbatim to maintain participant voices and context. Thematic analysis [37] offered a flexible and versatile method for qualitative analysis, making it well-suited for exploring concepts from Bourdieu's social theory. Initially, CR and AB independently immersed themselves in the interview data, identifying recurring themes and subthemes without imposing a theoretical framework. The initial focus was around unpacking and describing the participants’ experience within the student-led clinic. As they explored the data in depth, they considered the social spaces in which the student and patients were working and their relationships within them, based on previous work [38, 39]. At this point, AB and CR realised the potential of Bourdieu's lens and PK , with expertise in this approach joined the research team. Bourdieu’s concepts of field (the clinic as a structured social space with rules and hierarchies), habitus (internalized dispositions shaping individuals' actions), and capital (different forms of influence and prestige), emerged as powerful "thinking tools" [40, 24] to illuminate potential underlying mechanisms that provided the conditions for sustaining the clinic. Consistent with a Bourdieuan approach, we augmented our focus group data with reading of the student patient management plans undertaken on the structured clinic record, and field notes taken by CR and AB at the interprofessional student debriefings after each clinic. We continued in a second phase of analysis where all three authors (CR PK and AB) used open coding by identifying segments of data that related to Bourdieu’s concepts of habitus, field and capital. We clustered the initial codes into broader categories. Following further discussions, the coding framework was further refined in relation to our research questions. By re-coding both the converging and conflicting potential mechanisms that gave rise to the conditions necessary for the clinic to function, we were able to provide causal explanation of the data [41]. Triangulation was achieved through various means. First, the diverse data sources – focus groups, management plans, and field notes – provided complementary perspectives on the clinic experience. Second, the collaborative coding process and discussions ensuring different researcher viewpoints were considered. Finally, we continuously assessed the fit between our data and Bourdieu's framework, ensuring theoretical coherence and validity. We claimed sufficient information power [42] given our focused aim (i.e. clinic participants’ views and experiences of the clinic), the richness of the dialogue in the focus groups and our use of Bourdieuan theory during data analysis. Data were managed and analyzed using NVivo qualitative data analysis software (QSR International, Version 14)

### Ethics statement

This study was approved by the University’s Human Research Ethics Committee (Approval No: 2018/209). Written consent for participation was obtained from all participants to enable us to include their data in this study.

### Team reflexivity

As researchers, our team brought diverse professional backgrounds and perspectives to the exploration of student-led interprofessional clinics. CR, a general practitioner, IPL program developer and medical education researcher, played a lead role in setting up the clinic. He provided valuable insights into the clinical context in debriefing the students following the clinic sessions, and led the writing. Both AB, who was also an IPE program developer and PK had non clinical educational research backgrounds. AB intentionally took the role of interviewer to mitigate potential biases and ensure that students and patients felt at ease sharing their experiences. PK, with expertise in realist theories, provided a complementary lens to the analysis, adding depth to our understanding of the complex mechanisms underpinning the clinic. Discussions at each stage of the research process between the authors face to face and by email encouraged reflexivity about the methodological implications of particular decisions made during the course of the project [43]. This conscientious self-reflection aimed to transparently recognize and navigate potential influences that our professional backgrounds and perspectives might exert on the interpretation of the collected data [44] Mindful of people first language in disability research, we acknowledge that in our setting healthcare recipients appear to prefer the term ‘patient.’ [45] Though we use that term in this paper, we recognise some readers will prefer one of client, consumer, survivor or service-user.

## Results

Our data illuminated the student-led clinic as a field of practice, where intersecting fields included a patient support group, an aged care clinical practice within a teaching hospital, and supervised interprofessional teaching practices. We considered the positions and dispositions and habitus of our study participants within the field of the clinic, shedding light on the various forms and amounts of capital that were brought, enriched and exchanged by the students and patient partners.

To answer our research questions we developed three broad themes from our data: Fostering Students’ disposition to interprofessional care, Capitalizing on collaboration and patient empowerment and Culture of mutual capital exchange. We provide extracts of the participant voices to support the interpretation.

### Fostering students’ disposition to interprofessional care

Students adjusted to their positions and relationships with peers in the clinic. They varied in the way they internalized their relationships and expectations for working in the clinic. Some students had previous experience of working in an interprofessional team using simulated patient cases in the earlier years of their degree. The students thus brought differing amounts of social and cultural capital, but their disposition or tendency to practice interprofessionally was changed through their practice (actions) in the clinic.

*In first year we got together – but we didn’t have a real patient. It was a pseudo scenario and then we had to come up with a plan and a video. .. at uni… nothing like this. I find this a lot more beneficial to actually see the patient, then you can come back and discuss the issues. It was a completely different experience in clinic when you are in that chair, seeing that patient and then leave that room and discuss with your colleagues as to how to best manage.*

(Student)

Students recognised the central role of the patient as partners in influencing their own disposition to practice interprofessionally. Students demonstrated a number of strategies to engage with their peers in a context of mutual trust around the common goal of influencing the outcomes of patient care.

*…for me, what I’ve learnt today is not – not just from a medication point of view, but it’s to be able to talk to all the other health care professionals and then that’s how we provide it better – the best care for the patient that is to combined all the aspects and then to – that’s an overall picture and then, just, try to improve that for the patient.* (Student)

Students’ prior dispositions to healthcare practice takes shape through diverse interactions within various contexts which are largely uni-professional. In the interprofessional student-led clinic, balancing the need of patients with the limits of their clinical expertise, encouraged students to share their social (who they know) and cultural capital ( knowledge and competence) with their peers. For example a physiotherapy student said:

*It really just shows how connected everyone is. I know that you (pharmacy student) deal with medicines and doctors and we deal more with the mobility and gait and all that stuff, but doing this you see, we’ve seen this impairment, they see a medication that affects that impairment and stuff, so, it’s really obvious how much everything ties into each other.* (student)

In summarising this theme, student interactions in the clinic revealed a diverse range of dispositions (tendency to act), all bound by a shared commitment to patient-centered interprofessional care. This collective habitus, which can be seen as the "presence of the past in the present" [46] reflects their gradual journey towards interprofessional collaborative expertise. The student’s habitus is shaped by the accumulation of various forms of capital – knowledge, competencies, and collaborative skills stemming from their past experiences and socialization into uni- and inter- professional healthcare settings [23]. It was evident that some students were at different stages in this journey, as they internalized their experiences with peers and patients, shaped by their personal expectations.

### Capitalising on collaboration and patient empowerment

Patients in the student-led clinic were important contributors of capital. Patients invested significantly in the clinic by physically attending, sharing their stories and goals, and exposing themselves to examination. They felt that participating in the clinic was a worthwhile investment because they gained new understandings of their own well-being. One patient noting *“….that’s how they help us. So we are benefiting by this trial”.* Some patients felt that they made a unique contribution to student learning. This can be seen as the symbolic capital they brought to the clinic allowing the students to see different stages of a disease, share goals for well-being and gain rich insights into the patients’ real world experience, including how they accessed their health and social care related networks.

*“they see symptoms which are not in the textbook. We could have symptoms which are completely different….. I think they would be more aware and because they’re seeing such a – a different diversity of Parkinson’s”* (Patient participant)

The patients valued the interprofessional team approach in the student-led clinic and contrasted this with their past experiences of largely uni-professional care. They appreciated that students from different health professions communicated with each other to provide patient-centred care plans, offering a glimpse of hope for the future of healthcare. Patients expressed frustration that their own health care team didn’t communicate with each other,

*“About having a team approach, you’re dealing with things like Parkinson’s. I mean I’ve got a good GP and a good specialist, but they don’t really talk to each other very often….and they all talk about how we had a team approach but the – I haven’t seen too many teams really.”* (Patient participant)

Patients’ emphasis on self-directedness reflected their internalized values (habitus) that prioritize independence and self-care (cultural capital), and support networks (social capital). While acknowledging potential benefits of collaboration and social capital exchange with the students, some patients expressed frustration with overzealous assistance, perceiving it as a threat to their symbolic power and autonomy. This highlights the importance of respecting individual preferences and tailoring support to empower patients through capital accumulation, while avoiding paternalistic attitudes and recognizing the value of their existing cultural capital.

*Another aspect that occurred to me are the benefits of talking to these young people today - I get really, really cranky if – when we’re at our group, and we’re sitting next to somebody who is sitting there, and their carer’s sitting here, and [fellow patient partner] goes to get his coffee – and they’re giving it to him. I say, “Put it down. Let him or her get up themselves.” Because we don’t (want to be ) be killed with kindness. We want our independence as long as we can have it - - - - and the only way we’re going to get it, is us looking after ourselves…. and not taking shortcuts.* (Patient participant)

Patients explained that they wanted a conversation with the students or to read their recommendations, with input from the academic clinicians.

*"I would love to be part of the (student ) team to see the results, so I think the only negative I can think of is not being able to be part of the outcome."* (Patient participant)

In summary, in the student-led clinic, patients contributed significant symbolic capital through their investment in telling their stories, self-exposure, and willingness to participate in examinations. They brought cultural capital to the clinic by providing real-world insights into their health experiences and social capital in sharing how they access health and social care networks. Patients feel empowered by the interprofessional collaboration in the clinic and contrast it with their experiences of multidisciplinary care outside the student-led setting. However, in this setting, patient engagement with the clinic is constrained by the university's restriction on students sharing care plans with patients.

### Culture of mutual capital exchange

Students reflected on their cultural capital i.e. what they know, and how they can apply and exchange this knowledge in the context of the student-led clinic. In this case, a pharmacy student was able to reflect on the coming together of various professional relationships within the clinic where various forms of "capital" such as prestige or resources are at stake. These resources were not only available for exchange with patients but also with their peers. Specifically, this student's cultural capital encompassing knowledge of medications and polypharmacy, becomes a source of social capital earning them respect and appreciation from their peers who might not have that depth of knowledge.

*..as a pharmacy student, it was definitely really helpful in seeing and interviewing a Parkinson’s patient and seeing the full picture and how complex things can get. A lot of the medications in particular, there was a lot of polypharmacy, there was a lot of side effects that overlapped, and it really helps you to see how complex the bigger picture is when you’re actually speaking to a real Parkinson’s patient.* (student )

This pharmacy student highlighted a conflict arising from distinct forms of cultural capital, specifically between the pharmacy student’s professional expertise and the priorities of the patient. The application of evidence-based guidelines by the pharmacy student in medication management, an area where healthcare professionals possess expertise, clashes with the patient’s individual sense of well-being and their priorities. Moreover these guidelines may also conflict with the priorities established by the other healthcare professions. To address these conflicts in practice, discussions were brought to the student interprofessional team meeting. Here the student group engaged in reflexive negotiation to arrive at a consensus for future actions.

The physiotherapy student’s disposition to learn from their interprofessional peers allowed them to achieve insights not covered in their own curriculum, but advantageous for patient care.

*I really appreciate the fact that you actually broke it down and actually said, this is for this, and, you know, this is to help them to reduce, you know, all that stuff. It was really good to actually find out more information about medications. I think that’s something that physios in general should know what to do.* (student)

Some students were concerned that they lacked sufficient depth of disciplinary knowledge of their peers from other health care professions. They wanted faculty to assist with this, rather than relying on their own agency to engage with both their peers and the patients for their learning. One student wanted more instruction and to be provided with *“a set of standardised questions*“ to ask each patient. Some felt they had no expert knowledge in medication, an important component of the management of Parkinson's and therefore worried they couldn’t meaningfully participate. This student recognizes their lack of medication knowledge compared to their clinical educator and peers, and expresses a desire to gain this capital through learning and engagement.

*…. when we read patient charts just to find out what medications they’re on and what to help, our educator, obviously they’ve been working in hospitals for a long time, so they know what each one does, but I don’t., I, kind of, sat there going, I don’t know what that is, and don’t know what that does but okay….if I see that next time on someone else’s chart then I’ll know, good. They’ve taken it. It’s good. I’m good to go treating them, but I wouldn’t know what it does, if that makes sense.* (student)

Medical students also valued the social capital that they exchanged with their interprofessional peers in the clinic. Interestingly, they were surprised at the value of the cultural capital they gained in working with the patients. This student values the symbolic capital of the clinic (authentic patient interaction and interprofessional collaboration) and how it contributes to their learning.

*…the last time I ever studied neurology was back in second year… so it’s just good to see, actual Parkinson patients. I saw one for the first time in a while, took a history and exam. Like … seeing the clinical signs was a bonus working with multidisciplinary team and seeing what each team does differently, and you know learning from one another.* (student)

All students needed to develop a shared language through which to exchange their capital. As an example, the use of healthcare profession specific abbreviations was constraining patient care and student learning. Feedback to their peers promoted the use of a common language in describing the patients’ problems in the management plans, and avoiding acronyms. This student highlights the importance of using a shared language within the clinic to avoid symbolic exclusion of patients and other team members who might not understand profession specific jargon.

*…. and most of the medications were quite similar anyway so that made more sense. So it was it’s like reading a report with a bunch of abbreviations though, that maybe you’ve never used before but it’s very common for someone else.* (student)

Patients were able to assess what kind of knowledge and skills the students brought to the clinic when acting in their uni-professional role. Yet recognising that some professions needed more interprofessional knowledge (cultural capital) at this point in their learning trajectory. This patient recognizes the students' growing expertise and offers understanding for their limitations, while contributing their own lived experience to the learning process.

*I had a couple of the physio and a couple of the pharmacy. The medical students, I did ask a couple of questions, but I think it’s a little bit too early in their learning curve to answer it directly and responsibly, because everybody’s symptoms, as we all know with Parkinson’s is totally different. We’re not all the same, and I think that’s where it becomes a really big issue for some people.* (patient participant)

Patients actively engaged with students, providing valuable insights, experiences, and perspectives related to their Parkinson’s. By sharing their knowledge, challenges, and reflecting on student recommendations during the consultation, patients contribute to the social and cultural capital of the students, fostering a collaborative and enriching learning environment whilst enhancing their personal knowledge about their disability and impairment.

*"These different clinicians [from various professions], they ask pertinent questions of us, things that perhaps, you might forget about, and then you sort of think to yourself, 'Oh yes. That’s sort of important, and that’s happened to me.'" (patient participant)*

As students were rapidly socialised into the field of the clinic, they became attuned to the complex dynamics of interprofessional collaboration. Upon entering the clinic, students brought with them diverse, often substantial social (connections) and cultural (expertise) capital. While they may not be familiar with their peers, they were aware of the interprofessional roles expected of them. Students recognised the rich symbolic capital invested in the clinic offering them opportunities to exchange capital with peers and patients. They valued the patients’ willingness to entrust their stories, their health and social care problems to the students. Thus, there was mutual recognition of an exchange of capital, with patients recognising the capital that students bring. Like-wise, the patients showed their commitment to student learning by sharing their cultural capital with the students.

## Discussion

### Summary of key findings

This study, guided by Bourdieu's theory of field (social context), habitus (learned behaviours and dispositions), and capital (valuable resources), explored the dynamics of a student-led clinic situated at the intersection of diverse educational and healthcare contexts. Three fundamental mechanisms crucial for the clinic's sustainability emerged; fostering students' disposition to interprofessional care, empowering patients through collaboration, and nurturing a culture of mutual capital exchange. These findings shed light on the conditions shaping interprofessional learning within the clinic, offering valuable insights into how various forms of capital (social, cultural, and symbolic) influence the sustainability of interprofessional collaboration in student clinics.

The habitus plays a crucial role in shaping students' dispositions (inclinations) and guiding their responses to the clinic's enablers and constraints of interprofessional working. Their habitus fosters trust and readiness for interprofessional practice amongst their peers. Patients, on the other hand, feel empowered in recognising the importance of their well-being goals and their symbolic power in influencing student learning. The findings highlight the importance of recognising and valuing diverse forms of capital brought by students and patients, as well as the equal relationship between them within the clinic’s field of practice. The shared culture of mutual entrustment between students and patients significantly influences students’ learning experiences and enriches their habitus.

Students, through socialization into the field of interprofessional practice in the clinic, leverage their cultural capital to gain access to other forms of capital from patients, peers, and supervisors. The common goal of patient care drives students to build their capital through collaborative partnerships. The strength, trust, and value of interactions within the clinic contribute to the overall amount and quality of generated symbolic capital.

A dynamic exchange unfolds within the student clinic as participants—students and patients—contribute a wealth of social, cultural, and symbolic capital, engaging with their ingrained tendencies and learned behaviours (habitus). This mutual investment builds not only trust in a shared goal of better patient outcomes, but also enriches everyone's dispositions towards interprofessional care. Notably, the patients' contributions of symbolic capital - their stories, trust, and hopes - hold the highest value in this field, fostering a unique power dynamic where traditionally hierarchical uniprofessional roles become equal interprofessional partners. [47]

### Comparison with existing theory and literature

Our research findings reflect the underlying principles of the dynamic interplay between field, habitus and capital in providing interprofessional healthcare [44]. While aligning in part with the work of Bonello et al [44] in taking a Bordieuan lens, our empirical study extends this perspective by illustrating a multiplicity of influences in the reproduction of a complex IPE activity, the student-led clinic. In our study there was no evidence of symbolic power struggles between the students, as predicted by other scholars reporting medical domination [28, 44, 48]. In clinical settings, IPE might be expected to create role conflicts in terms of certain professions such as medical students struggling to see the benefit of the IPE experience [49]. Students in our study acknowledged professional boundaries and mutually respected the overarching goal of patient care. The observed struggle was not centered on perceived disparities in cultural or social capital but rather on learning to collaborate effectively for patient-centred healthcare [50]. The central role of the patients appeared to disrupt the traditional power relations as patients did not favour the capital of one healthcare profession over another.

Our data supports the role of habitus serving as a framework, illustrating how the clinic’s operation shaped students' enduring dispositions (tendencies, attitudes, and capabilities) to engage in interprofessional practices. Informed by their habitus, students creatively navigate the constraints and enablers of becoming interprofessional [51]. By emulating authentic interprofessional practice tailored to patient needs, students negotiated with the diverse perspectives of peers, patients and supervisors within the clinic. The evolution of students’ habitus emerges as a ‘practice-unifying and practice-generating principle,’ [46] significantly influencing students’ readiness and trust in interprofessional practice. Notably the habitus concept resonates to some extent with the idea of an interprofessional identity evolving from a uniprofessional identity [52].

The patients’ perception of their symbolic power stemmed from a realistic understanding of what could be achieved for their well-being. This emphasized the importance of connections (social capital) between students and patients, creating a source of influence that leads to the accumulation of shared symbolic value, encompassing cultural and social aspects [24]. Within the clinic’s dynamic, power seemed to be culturally and symbolically created and constantly reinforced through the interactions among team members. Trust played a crucial role, fostering the willingness of both students and patients to share their cultural capital [22].

Sharing cultural capital emerged as an important practice-generating principle, promoting a sense of collective identity. Individuals’ practices or actions are the consequences of their cultural capital and habitus interacting within the context of a given field [53]. Being able to convey professional knowledge regarding the situation of a patient and consider the professional views of others is essential to interprofessional work, as a means to providing the best patient outcomes [54, 50]. Positive experiences of working interprofessionally in group settings can lead to increased trust in other professional groups in the workplace, while the benefits of this collaboration continue to build over time [55]. Our findings support the notion that to sustain interprofessional student-led clinics, the IPE leaders must persuade a diverse range of stakeholders, not only those who would be engaged as learners, supervisors, and administrators but those with the power to provide diverse forms of capital and economic resources [8].

### Methodological strengths and challenges

The utility of using Bourdieu's theory lie in its ability to provide a multi-dimensional understanding of the clinic and to uncover the underlying mechanisms that sustained or constrained interprofessional working in a student led clinic.

A strength of this study is its extension of the existing literature in three ways. First, it provides insights into the experiences of two key stakeholder groups, prospective healthcare practitioners and patients living with a chronic disability on the perceived value of a student-led clinic. Second, using Bourdieu offers a theoretical framework that goes beyond economic considerations and emphasises the importance of alternative forms of capital in promoting interprofessional practice. Third, it replaces the idea of a power struggle between medicine and the other healthcare professions causing a lack of collaboration to one of cooperation between patients and the student team around patient well-being goals.

In terms of the challenges, we did not employ the range of Bordieuan methodologies, such as looking at our field in relationship to other fields; in particular the recognized fields of power in the university and the health care providers. We do not have the data from the multiple clinicians and educators consulted in setting up the clinic. We acknowledge that in applying Bourdieu's theory there were some methodological challenges in operationalizing the concepts of field, habitus, and capital. Despite the limitations of our sample, being within one institution, and amongst students and patients who volunteered and may have had more positive dispositions to engage with IPE, we still believe the findings will be valuable to educators wishing to develop their own clinics or reflect on their own established practices.

### Implications for educational practice

Interprofessional education (IPE) student-led clinics offer compelling avenues for fostering collaborative healthcare practice. Our findings suggest ways of optimising IPE student and patient experiences by facilitating the investment in and exchange of differing forms of capital to build sustainable clinic models. There is collective symbolic power in the accumulation of various forms of ‘capital’ in shaping interprofessional practice. Cultivating trust and open communication within this system becomes the catalyst enabling students and patients and educators to co-create a vibrant interprofessional learning environment.

In order for student clinics to thrive in a sustainable fashion, educators must shift their focus beyond solely maximizing financial resources. Instead, they should champion investments in a wider range of capital forms. This requires active participation from all stakeholders – faculties, patient partners, service providers, and students – each contributing their unique strengths and expertise. Inter-organizational partnerships, such as university-hospital-community collaborations, and student-patient co-design projects, become critical pathways for leveraging the collective symbolic power of the clinic.

**Fig. 1:**
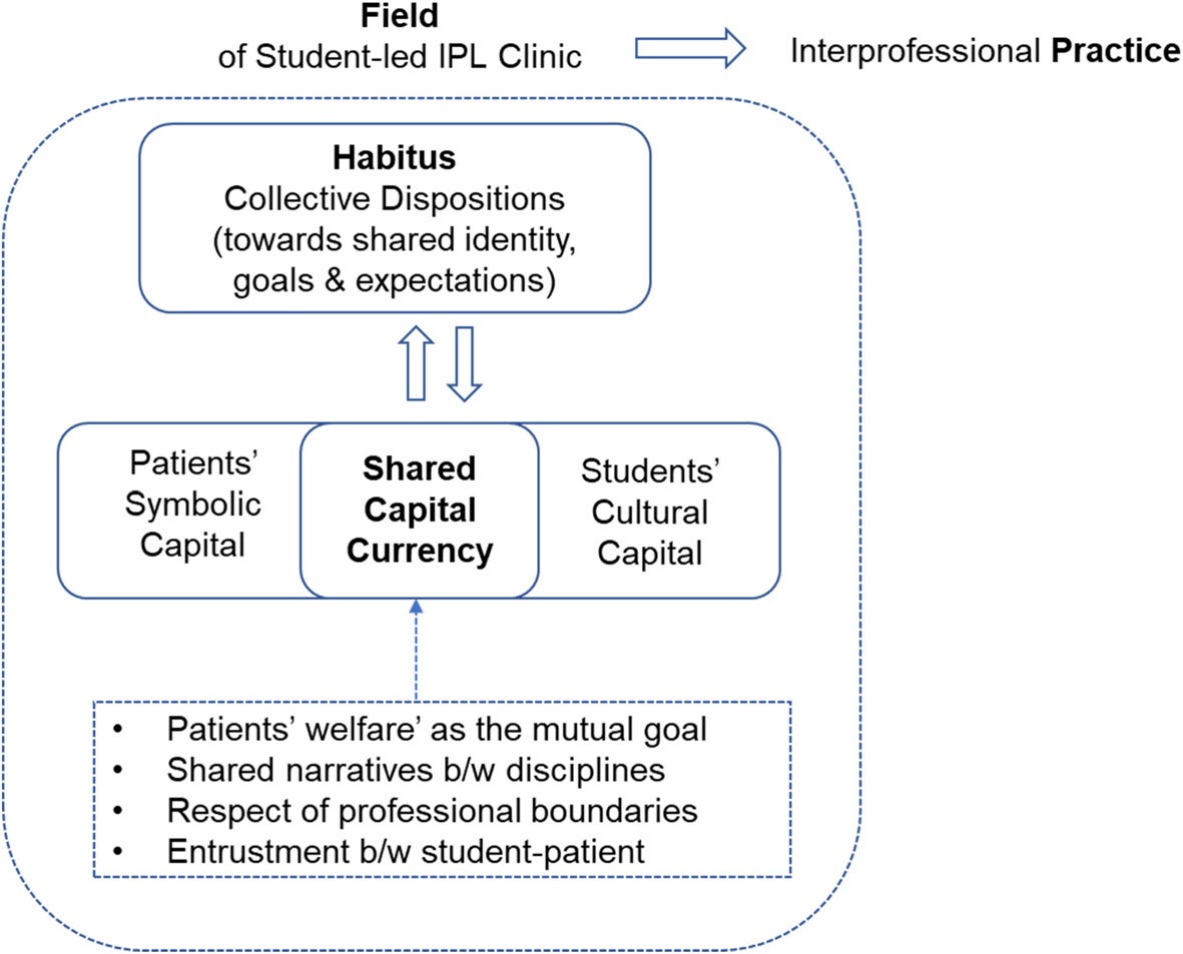
A framework for conceptualising Interprofessional practice in a student-led clinic though the Bourdieuan concepts of field, habitus and capital

Complexity based logic models may provide a useful evaluation process for tracking how different forms of capital translate into tangible outcomes for students, patients, educators and the wider healthcare system [17]. A framework for conceptualising Interprofessional practice in a student-led clinic though the Bourdieuan concepts of field, habitus and capital is given in Fig. 1.

### Implications for further research

Given the methodological insights and constraints associated with this study, further research is advised employing sociological methodologies such as ethnography from the outset of studies to further elucidate the opportunities and struggles in setting up IPE clinics and their impacts on patient outcomes and student professional development. We encourage researchers to explore the impacts of different types of Interprofessional student-led clinics e.g., vaccination clinics, pre-diabetic care, or rehabilitation clinics. We suggest further research is needed to explore how to effectively manage the power dynamic of physician dominance in other student led clinic contexts. Further research could also consider the adaptability of the current findings in other settings in both resource rich and resource challenged countries. By reframing the research question, a Bourdieuan approach may help to explain how various entities within the university faculty leadership groups, the health care educators from differing professions, leadership groups from within healthcare providers, and patients’ representation could exercise their powers and generate mechanisms to make student-led interprofessional clinics flourish.

## Conclusion

This study addresses an important gap in theoretically informed investigations of the sustainability of student-led clinics, using Bourdieu’s theory. Going beyond economic considerations, our findings emphasize three interconnected mechanisms that offer nuanced insights for providing the conditions for sustainable development of student led clinics. First, student dispositions towards patient-centered interprofessional healthcare, evolved through interactions with each other and with patients. Second, their shared habitus, reflects a gradual journey toward collaborative expertise, shaped by accumulated social and cultural capital. The recognition of patients as equal partners in bringing their own forms of capital significantly influences clinic dynamics. Third, the exchange of diverse forms of capital, in alignment with Bourdieu's theory, optimizes participants' experiences, enriching the habitus of both students and patients. These findings highlight the importance of optimizing various forms of capital and embracing patients as dynamic contributors for the student-led clinic to flourish.

## Data Availability

Datasets supporting the conclusions of this article are included within the article. The datasets generated and analysed during the current study are not publicly available due to confidentiality agreements approved by the Human Research Ethics Committee but are available from the corresponding author on reasonable request.
